# The Hospitals' Future in the Balance

**Published:** 1920-11-06

**Authors:** 


### The Hospital

The Workers' Newspaper of Administrative Medicine and Institutional Life, Administration, National Insurance and Health.

No. 1796, Vol. LXIX. SATURDAY, NOVEMBER 6, 1920. PRICE TWOPENCE

### THE HOSPITALS' FUTURE IN THE BALANCE.

The Government hope that about the end of this week it will be possible to get ahead with the Ministry of Health (Miscellaneous Provisions) Bill. Dr. Addison has been wise to meet Members of Parliament specially interested and to explain to them the powers that are sought to enable local authorities to contribute to local voluntary hospitals. We stress the fact that the voluntary hospitals, which would obtain the questionable benefit (the last word being used in its Parliamentary sense), are, as the Bill stands, local in the sense that they are for the purpose yoked to the geographical authority to which their situation attaches them. The hospitals of the great cities and all the special hospitals are (possibly to their benefit in the dictionary sense) out of the Bill altogether, as they would have little or no claim upon the local authority unless a County Council is nominated by statute as the local authority.

Dr. Addison again assured his fellow Members of Parliament that there is no sinister conspiracy to put the hospitals on the rates. His main preoccupation is that these institutions are insufficient for the needs of the country, their total number of beds being 45,000, while there are 39,000 beds in fever and small-pox hospitals, and 94,000 hospital beds in Poor-law infirmaries. He cannot think of the needs of the public *qua* hospitals in the terms of 45,000 beds, but has in mind the entire hospital accommodation throughout the country. The Minister went on to speak in appreciative terms of the labours of voluntary, medical, and lay workers, and said that no Minister of Health in his senses would set out to destroy the voluntary hospitals. While no absolutely definite statement to the effect was made, Dr. Addison's remarks can be construed to convey that although local authorities must have a determining voice in the way their money is spent on general hospitals instituted by them, he does not claim that they should interfere with the hospitals of the voluntary service, to which they merely subscribe. We are aware that in this respect the Minister's remarks have been interpreted differently, but from the very full report before us we cannot agree that municipal interference in the management of voluntary hospitals is at the present stage foreshadowed.

The main point, or, as Sir Napier Burnett calls it, the plain issue, is how in the future the people of this country are to be mended of their ailments? As the same authority says, "The achievement of that end is clearly of more importance than a Parliamentary victory for the voluntary principle or for municipalisation, or any other abstract argument whatsoever." If, rate and tax harried as we are, we can put cost aside, or provisionally agree that if needs must we are willing to pay twice as much through one service as we would through the other, we must from the economical standpoint alone adopt the axiom that health is wealth, and the best means to achieve it are the least costly in the long run. But we must be satisfied as to what are the best means, and few of us so far in this respect have been converted by Dr. Addison's crusade.

Concurrently with the present issue of this journal a Conference of Medical and Allied Societies is sitting to consider the hospital clauses of the Bill. This conference is representative of many interests—of doctors, dental surgeons, pharmacists, nurses, midwives, and others who will be intimately associated with any health scheme—and, as one learns from the agenda, is a body which examines the subject from the point of view of the ratepayer as well as that of the professional worker. The views of such gathering on this matter of vital public importance will be thoughtfully considered by all who are concerned, and those, if they knew it, are the public at large. What those views will be is for the moment a closed book, of which we do not in anticipation attempt to lift the cover. But we may speak of our ideas and our ideals.

The veil over possibilities is lifted a little by the offer made by King's College Hospital to staff and conduct the medical side of the Camberwell Poor-law Infirmary. The physicians and surgeons will make no charge for their work at the infirmary, and the students of the hospital will under them secure wider fields of study. Thus the infirmary gains the services of a consultant staff and the hospital adds to its teaching field. Naturally, a condition that the medical staff shall have full medical control is required. The position of the senior man, usually to be found as a medical superintendent at an infirmary, may be affected; but that point, tactfully dealt with, is but a matter of detail. The possibility of those hospitals which are still called Poor-law infirmaries being managed by the voluntary and older service has been already suggested by Sir Arthur Stanley and others of our leaders. The possibility could conceivably become a fact, and its consideration is one of the lines that our reflections should pursue at the present juncture. That such a system would be popular, if popularity were all, we do not doubt; that it would be sound policy we are equally sure.

The voluntary hospitals, to use a shop-keeping simile, do not dress their windows skilfully. They are prone to keep the best goods in the background. Their chief functions of teaching, of consultant service and of preventive work are too often obscured by masses of statistics of gigantic aggregates of attendances of out-patients and scrappy facts as to the huge quantities of pills and ointments that pass through the dispensary hatches. The public are made aware of what is being done for the patient of to-day, and are left to remain ignorant of what is being done for the patient of to-morrow, and for the men and women who because of the voluntary hospital service will never become patients at all. The public, properly enlightened, would respond generously, and it is quite appropriate that the State, without recourse to a Ministry of Health (Miscellaneous Provisions Bill), could adequately provide for public services in respect of teaching, of medical students and nurses, health and social workers, and the like, and of the pursuit of preventive medicine and research already in the hands of men whose ability could not be surpassed in any other service.

But the process will not be a cheap one. Few good things are cheap. The present day, when voluntary hospitals are starving themselves for want of means to procure adequate scientific and hygienic sustenance, must pass. It will not be a cheap service, but it will not in our opinion be so costly, not merely in respect of financial considerations, as would be the hospital service which is such an important feature of Dr. Addison's Bill.

